# Characterization of the antiproliferative activity of Xylopia aethiopica

**DOI:** 10.1186/1747-1028-7-8

**Published:** 2012-03-12

**Authors:** Aphrodite T Choumessi, Mathieu Danel, Stefan Chassaing, Isabelle Truchet, Véronique B Penlap, Anatole Constant Pieme, Tazoacha Asonganyi, Bernard Ducommun, Annie Valette

**Affiliations:** 1 Université de Toulouse; ITAV-UMS3039, F-31106 Toulouse, France; 2 CNRS; ITAV-UMS3039, F-31106 Toulouse, France; 3 CHU de Toulouse, F-31059 Toulouse, France; 4 Department of Biochemistry, Faculty of Science, University of Yaoundé I, B.P. 812, Yaoundé, Cameroon; 5 Department of Physiological Sciences, Faculty of Medicine and Biomedical Sciences, University of Yaoundé 1, Yaoundé, Cameroon; 6 Université Toulouse; LSPCMIB-UMR5068, F-31062 Toulouse, France; 7 Centre Pierre Potier, ITAV-UMS3039, 1 place Pierre Potier, B.P. 50624, F-31106 Toulouse Cedex 1, France

**Keywords:** Xylopia aethiopica, ent-15-oxokaur-16-en-19-oic acid, cytotoxicity, DNA damage

## Abstract

**Background:**

Xylopia aethiopica, a plant found throughout West Africa, has both nutritional and medicinal uses. The present study aims to characterize the effects of extracts of this plant on cancer cells.

**Results:**

We report that X. aethiopica extract prepared with 70% ethanol has antiproliferative activity against a panel of cancer cell lines. The IC50 was estimated at 12 μg/ml against HCT116 colon cancer cells, 7.5 μg/ml and > 25 μg/ml against U937 and KG1a leukemia cells, respectively. Upon fractionation of the extract by HPLC, the active fraction induced DNA damage, cell cycle arrest in G1 phase and apoptotic cell death. By using NMR and mass spectrometry, we determined the structure of the active natural product in the HPLC fraction as ent-15-oxokaur-16-en-19-oic acid.

**Conclusion:**

The main cytotoxic and DNA-damaging compound in ethanolic extracts of Xylopia aethiopica is ent-15-oxokaur-16-en-19-oic acid.

## Background

Natural compounds have attracted considerable attention as preventive and therapeutic agents against cancer [1]. Indeed, 74% of anticancer compounds are natural products or their derivatives. According to the World Health Organization (WHO), 80% of the population in Africa and in some Asian countries still use plant preparations to treat their illnesses, including cancer. The cloves of the plant Xylopia aethiopica, a member of the custard apple family, Annonaceae, are used as a spice in various traditional dishes of Western and Central Africa. The plant is also used in decoction to treat dysentery, bronchitis, ulceration, skin infection and female sterility. Several studies have shown that X. aethiopica extracts possess antibacterial [2-5], antifungal [6] and anti-plasmodial [7] activities. X. aethiopica extract contains an antioxidant activity [8]; it also increases antioxidant defense and protects rats from the adverse effects of irradiation [9,10].

Although some extracts of this plant have antioxidant properties, others have cytotoxic effects on a wide range of cancer cell lines [11]. A recent study of various Cameroonian spices [12] found that extract of X. aethiopica had cytotoxic activity against pancreatic and leukemia cells sufficient for the plant to be considered a potential source of cytotoxic compounds, according to the plant-screening program of the National Cancer Institute. In this report, we confirm the cytotoxic activity of X. aethiopica extract against a panel of cancer cell lines and identify the main compound responsible for this cytotoxic effect: ent-15-oxokaur-16-en-19-oic acid (EOKA). Furthermore, we show that EOKA triggers DNA damage and accumulation of the cells in the G1 phase of the cell cycle, followed by apoptosis.

## Methods

### Cells

Human cancer cell lines HCT116 (from a colorectal cancer), U2OS (from an osteosarcoma) and SUM-159PT (from a breast carcinoma) were cultured in DMEM. The human pancreas adenocarcinoma cell line Capan-2 and normal human skin primary fibroblasts (kindly provided by P. Descargues, Toulouse) were cultured in DMEM F-12. Leukemia cell lines KG1a and U937 were grown in IMDM. All culture media were supplemented with 10% foetal calf serum, 100 u/ml penicillin and 100 μg/ml streptomycin.

### Extraction of X. Aethiopica

Dried fruits of X. aethiopica were collected from spice vendors and the voucher specimen was identified at the Cameroon National Herbarium. Dried fruits were ground and extracted with 70% ethanol (100 mg/ml) for 2 h at 55°C with shaking every 30 minutes. The suspension was centrifuged at 4000 rpm for 10 minutes at 25°C and the resulting supernatant extract was filtered and stored at -20°C.

### Stability assessment of the dried residue by LC/MS

Analytical liquid chromatography-mass spectrometry (LC/MS) was carried out using Waters equipment: an Xbridge C18 column (4.6 × 150 mm; 5 μm particle) for separation, a Waters 2996 photodiode array detector and a Waters 3100 mass detector (ES mode) for detection. Compounds were eluted with a gradient of water and acetonitrile containing 0.05% of formic acid at a rate of 17 ml/min. Ten microlitres of sample solution were injected for each analysis.

Each LC/MS analysis was performed by injecting 500 μl of the X. aethiopica extract diluted with 500 μl methanol. To check the stability of the extract, 20 ml was dried under vacuum and the dried residue was stored at room temperature for a week; it was then solubilized in a mixture of 20 ml of methanol and 2 ml of DMSO and analyzed by LC/MS. The resulting chromatograms were identical, thus we conclude that the dried residue is stable at room temperature.

### HPLC fractionation of X. Aethiopica extract

Fractionation by HPLC was carried out using a Waters Xbridge C18 column (19 × 150 mm; 5 μm particle), a Waters 2996 photodiode array detector and a Waters 3100 mass detector (ES mode) for detection. Compounds were eluted with a gradient of water and acetonitrile at 1 ml/min. For each fractionation run, we injected 350 μl of the solubilized residue in methanol/DMSO as previously described.

### Antiproliferative effect

Cell proliferation was assessed first by measurement of viable cell number. HCT116, U2OS, SUM-159PT, Capan-2, U937, KG1a and normal fibroblasts were seeded in six-well plates (5 × 10^4 ^cells/well). Cells were treated 24 hours later with X. aethiopica extract (25 μg od dry powder per ml). After 4 days, the number of cells in each well were counted on a cell counter.

The effect of X. aethiopica extract on cell proliferation was also examined with the colorimetric reagent WST-1 (Roche). HCT116 cells were seeded into 96-well plates (3 × 10^3 ^cells/well). After 24 h, increasing concentrations of X. aethiopica extract or fractions obtained by HPLC were added (six wells for each concentration). After 4 days, cell viability was estimated according to the manufacturer instructions, by measuring the optical density of each well at 450 nm with a microplate reader (Labsystems Multiskan).

Statistical tests were performed using Excel and PRISM softwares.

### Flow cytometry analysis

HCT116 cells were seeded in 60 mm plates (3 × 10^5 ^cells/plate) then treated 24 hours later with HPLC fractions of X. aethiopica extract. Cells were harvested after various times of treatment, fixed in ice-cold 70% ethanol, washed with PBS containing1% bovine serum albumin, and permeabilized with 0.25% Triton X-100 for 10 minutes. Cells were stained with a mouse monoclonal antibody against phosphorylated γ-H2AX (diluted 1/1000; Upstate Biotechnology) followed by an Alexa 488-conjugated anti-rabbit antibody (diluted 1/500;). DNA was stained with propidium iodide (10 μg/ml) in the presence of RNAase (10 μg/ml) for 1 h at room temperature. The cell cycle phase and presence of γ-H2AX staining in individual cells was determined by flow cytometry analysis (Accuri C6 cytometer, Cflow plus software).

### DAPI staining

U2OS cells were seeded in six-well plates (5 × 10^4 ^cells/well) containing 12 mm coverslips and subsequently treated for various times with peak 1 (20 μg/ml) or with doxorubicin (10 mM). Cells were fixed with 4% formaldehyde (Diapath), permeabilized in 0.1% (w/v) Triton X-100 for 5 min, washed in PBS and stained with DAPI.

### Western blot analysis

U2OS cells were treated for various times with peak 1 (20 μg/ml). Cells were washed with PBS and lyzed in ice-cold lysis buffer (Tris-HCl pH 7.5, 250 mM NaCl, 5 mM EDTA, 1 mM DTT, 0.1% Triton X-100, 50 mM sodium fluoride, 1 mM sodium orthovanadate, 100 μg/ml PMSF and TPCK, 50 μg/ml TLCK, 2 μg/ml leupeptin, 1 μg/ml pepstatin and 1 μg/ml aprotinin). Proteins (100 μg) were separated by SDS-polyacrylamide gel electrophoresis and transferred to nitrocellulose membranes by semi-dry blotting. Membranes were hybridized with antibodies against PARP (diuted 1/500; BD Pharmingen), p21 (antibody sc-397, diluted 1/2000) and p53 (antibody sc-126, dilted 1/2000) (both from Santa Cruz Biotechnology,). To control for equal loading, the western blot was probed with an antibody against actin (diluted 1/10000, Chemicon,).

### Identification of the cytotoxic natural product

The chemical structure of the cytotoxic natural product (peak 1 of the HPLC profile) was determined by mass spectrometry (positive and negative electrospray ionization) with a Waters 3100 mass detector and by NMR analyses. ^1^H and ^13 ^C spectra NMR spectra were carried out in either methanol-d_4 _or deuteriochloroform as solvents and were recorded with a Brucker Avance 300 spectrometer at 300 and 75 MHz, respectively. Carbon multiplicities were determined by either DEPT135 or J_mod _experiments. Diagnostic correlations were determined by two-dimensional NMR correlation spectroscopy (COSY), heteronuclear multiple-quantum correlation spectroscopy (HMQC) and heteronuclear multiple-bond correlation spectroscopy (HMBC).

## Results and discussion

**Figure 1:**
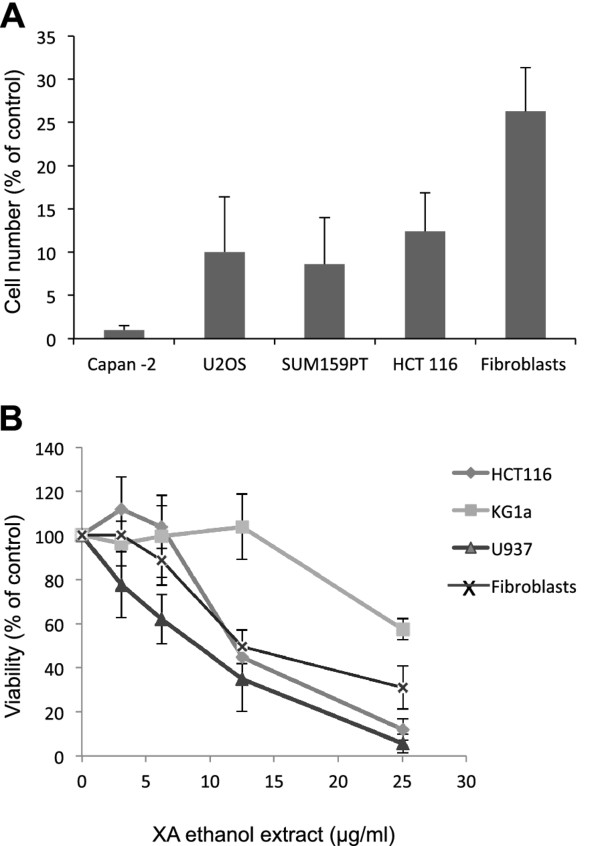
**The antiproliferative effect of extracts of X. aethiopica on various cancer cell lines**. (**A**) The indicated cell lines were treated with either 70% ethanol (control vehicle) or 25 μg/ml X. aethiopica extract for 4 days. Cell numbers were counted and normalized to the control value. Student's t-test gives a p-value < 0.005 for all cell lines. (**B**) Dose-response curves for the effect of X. aethiopica extracts on the viability of HCT116, KG1a, U937 cells and normal fibroblasts. Cells were treated with various concentrations of X. aethiopica extract for 4 days and cell viability was determined using a WST-1 assay (triplicate determination). The values are the means of three independent experiments and the error bars represent the standard deviation.

We first evaluated the cytotoxic effect of four days continuous treatment with 25 μg/ml of X. aethiopica extract in 70% ethanol on proliferation of various cancer cell lines (Figure 1A). This treatment produced a highly significant (p < 0.005) decrease in the number of cells in all the lines we tested. The effect on normal fibroblasts was less pronounced: 30% of cells survived the dose that fully inhibited cancer cell line growth. We next used a colorimetric cell proliferation assay with the reagent WST-1 to determine the dose-response effect of X. aethiopica extract on HCT116 colon cancer cells. The extract induced a dose-dependent inhibition of cell viability (IC50, 12 μg/ml; Figure 1B). We also determined the effect of X. aethiopica extract on survival of immature (KG1a) and mature (U937) acute myeloid leukemia cells. Although the extract decreased the survival of both cell lines, the immature line KG1a (IC50, > 25 μg/ml) was more resistant than the mature U937 cells (IC50, 7.5 μg/ml). Since KG1a leukemia cells have increased ability to carry out DNA repair [13], our data suggested that DNA repair might play a crucial role in mitigating the cytotoxic effect of X. aethiopica.

Our data indicate that X. aethiopica extract is cytotoxic to cells from a variety of cancers: carcinoma, osteosarcoma and leukemia. These data confirm and extend those of Kuete et al. [12], who reported that, among the 34 Cameroonian spices and plant tested, a crude extract of X. aethiopica was one of the most cytotoxic against MiaPaCa-2 pancreatic cancer cell line and CCRF-CEM leukemia cells. Another study characterized the antiproliferative effect of X. aethiopica extract on human cervical cancer cells [14].

**Figure 2:**
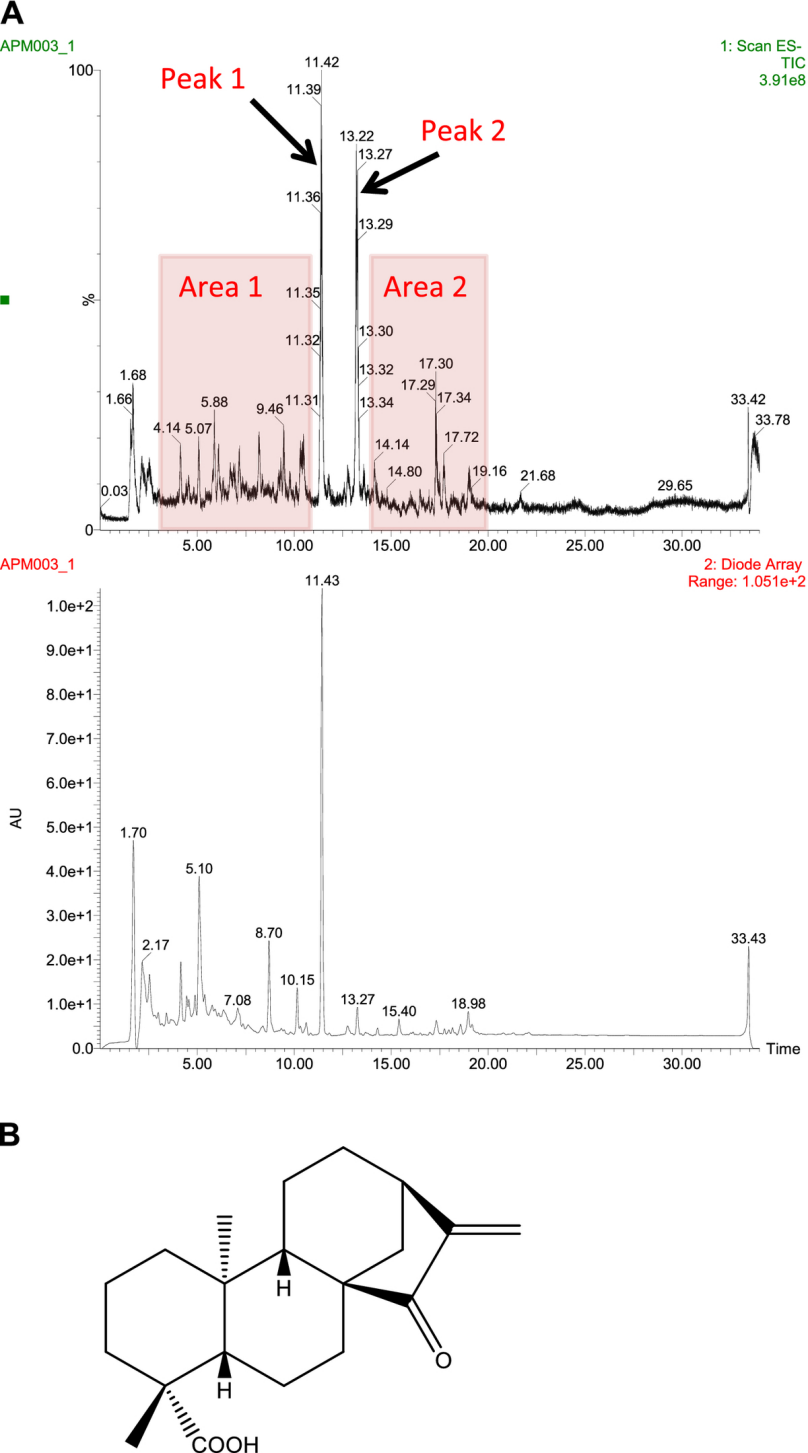
**LC/MS fractionation of X. aethiopica extract and structure of EOKA, the active compound in peak 1**. (**A**) High-performance liquid chromatography (C18 HPLC column) profile of 70% ethanol extract (ES-TIC: mass 100 to 600, and UV: diode array 210-400 nm). Selected areas 1 and 2, and peaks 1 and 2 are indicated. (**B**) Chemical structure of ent-15-oxokaur-16-en-19-oic acid (EOKA).

Considering the potency and relative selectivity of X. aethiopica extract for cancer cells, we decided to characterize further the cytotoxic activity in the extract. To this end, we analysed the extract by LC/MS (Figure 2A). From the chromatogram, we identified four fractions of interest: area 1 (retention time, 3-11 min), peak 1 (retention time, 11.4 min), peak 2 (retention time, 13.2 min) and area 2 (retention time, 14-20 min). We then isolated these fractions at a preparative scale by fractionating 2 ml of the residue solubilized in methanol/DMSO.

**Figure 3:**
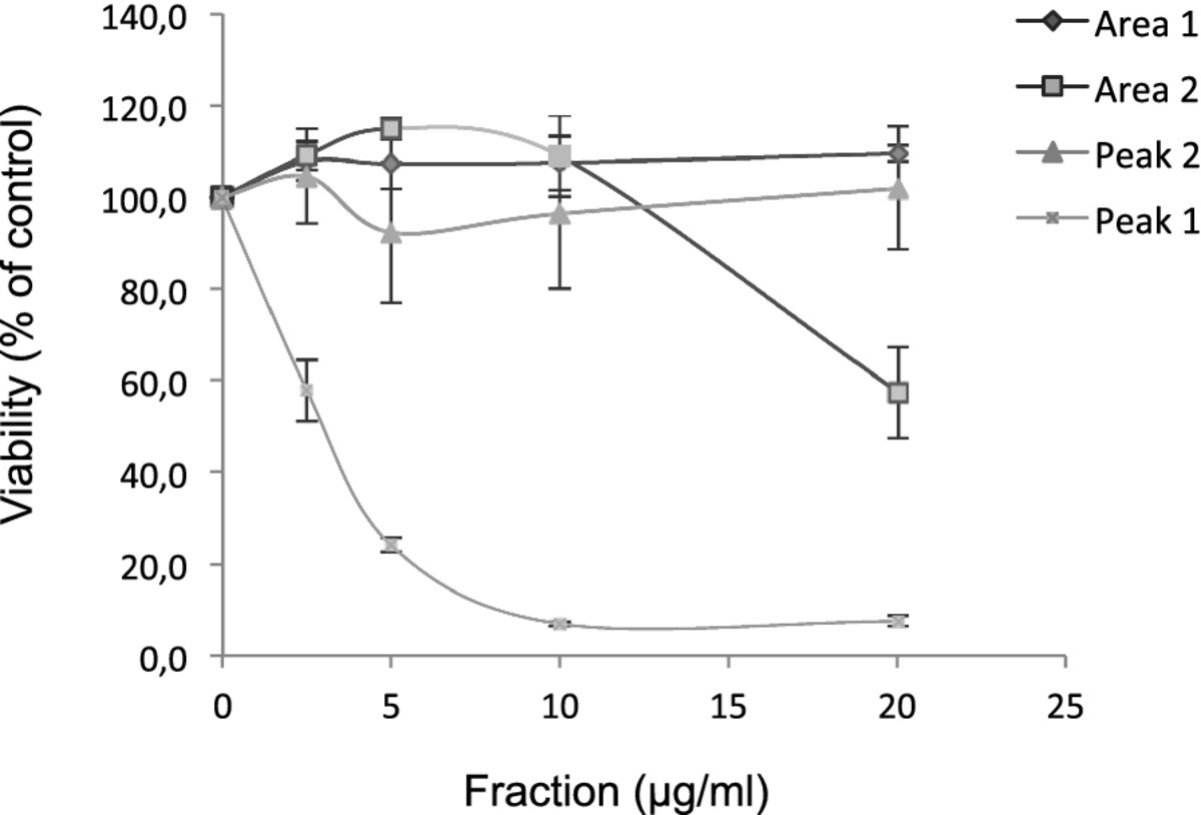
**The antiproliferative effects of various LC/MS fractions of X. aethiopica extract**. Dose-response curves showing the effects of the four fractions of X. aethiopica extract obtained by HPLC (Figure 2) on HCT116 cell viability. Cells were treated for 4 days with increasing concentration of the various fractions (area 1, area 2, peak 1, peak 2) and cell viability was determined by WST-1 assay (triplicate determination). The values are the means of three independent experiments and the error bars represent the standard deviation.

We assayed the cytotoxicities of the four selected fractions (i.e., area 1, area 2, peak 1 and peak 2) against HCT116 cells (Figure 3). Area 1 and peak 2 were devoid of cytotoxic activity and area 2 had only a slight cytotoxic effect at high dose (20 μg/ml). By contrast, peak 1 exerted a cytotoxic effect at low dose (IC50, 3 μg/ml). Thus, peak 1 was selected for further study.

**Figure 4:**
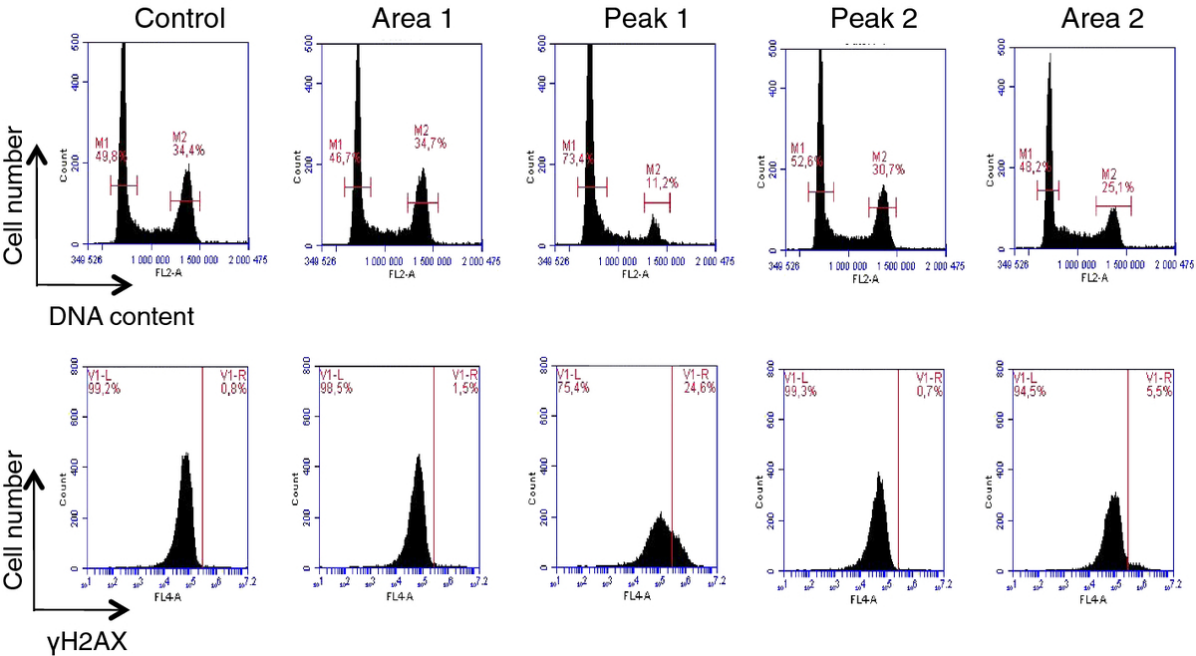
**The effect of peak 1 on DNA damage and the cell cycle**. Flow cytometry analysis of DNA content (top panel) and γH2AX expression (lower panel) in U2OS cells after treatment with fractions of X. aethiopica extract. The cells were treated for 24 h with 20 μg/ml of the various fractions. The percentage of cells with G1 and G2/M DNA content are indicated. The percentage of γH2AX- positive cells above the threshold line indicates the amount of DNA damage.

Many of the commonly used cancer chemotherapeutic drugs exert their cytotoxic effects by inducing DNA damage, which activate cell cycle checkpoints that coordinate cell-cycle progression with DNA repair [15]. To determine whether the cytotoxic effect of peak 1 was due to DNA damage and activation of cell cycle checkpoints, we used biparametric flow cytometry analysis of DNA damage and DNA content in U2OS cells. We used immunochemical staining for histone H2AX phosphorylation at Ser139 (γ-H2AX) as a measure of DNA damage and propidium iodide staining to monitor DNA content of control cells and cells treated with 20 μg/ml of peak 1, peak 2, area 1 and area 2 (Figure 4). An increase in H2AX phosphorylation was detected by the presence of cells with higher signal from γ-H2AX than in control cells. Peak 1 induced accumulation of cells with more DNA damage than control cells, whereas no significant change in the proportion of cells with DNA damage was observed after treatment with area 1, area 2 or peak 2. Indeed, whereas less than 1% of control cells presented DNA damage, 25% of peak 1-treated cells had damaged DNA. Furthermore, our results show that peak 1 DNA damaging activity was associated with an accumulation of U2OS cells in the G1-phase of the cell cycle.

**Figure 5:**
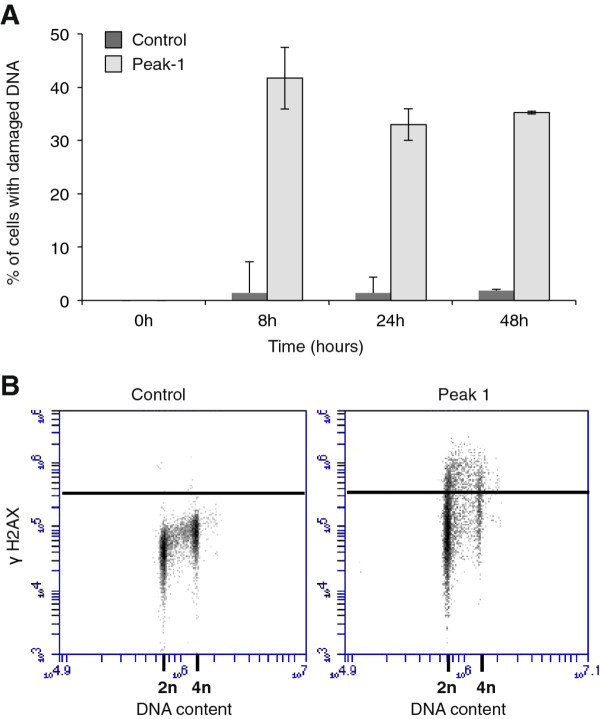
**The kinetics of peak 1-induced DNA damage**. (**A**) DNA damage in treated with peak 1 (20 μg/ml) or with for various times up to 48 h. Bars represent the mean of two independent experiments. (**B**) Bivariate analysis of DNA damage (γH2AX) and DNA content (2n, 4n) after treatment with 20 μg/ml of peak-1 for 24 h.

To characterize in more detail peak 1-induced DNA damage, we performed a kinetic study (Figure 5A). DNA damage could be detected within 4 h after treatment of with peak 1. We also performed a bivariate analysis of DNA content and γ-H2AX staining after 24 h treatment with peak 1 (20 μg/ml) (Figure 5B).

Screening of some medicinal plants commonly used in Nigeria found that X. aethiopica had mutagenic activity [16]. This mutagenic activity could be due to the DNA damage we found induced by peak 1 in the X. aethiopica extract. raising the issue of the use of this medicinal plant as spice. By contrast, other studies have found that X. aethiopica extract protects rats against the adverse effects of γ irradiation [9,10], suggesting that X. aethiopica extract contains both DNA damaging agents and protecting agents against the DNA damaging effect of irradiation.

**Figure 6:**
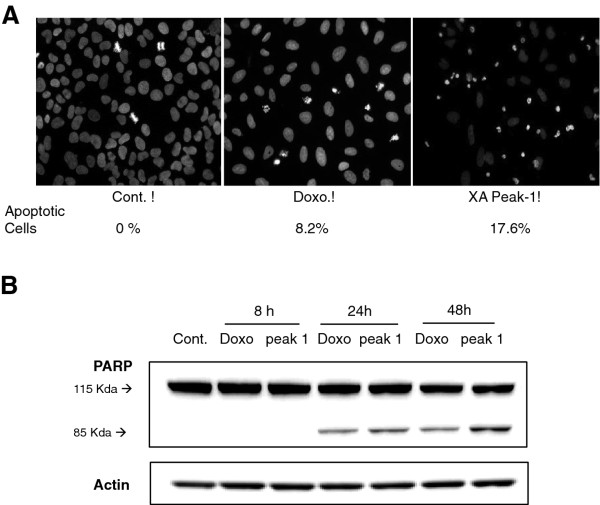
**The apoptosis-inducing effect of peak 1**. (**A**) DAPI staining of U2OS cells after 24 h treatment with peak 1 (20 μg/mL) or 10 nM doxorubicin. The images are representative of three independent experiments. The mean percentage of apoptotic cells is indicated. (**B**) Western blot analysis of PARP after treatment of U2OS cells with peak 1 (20 μg/ml) or doxorubicin (10 nM) for 8, 24 and 48 h. Full-length 115 kDa PARP and its 85 kDa proteolytic fragment are shown. Actin is presented as a control for equal loading.

It has previously been reported that X. aethiopica fruit extract induces cell death by apoptosis in the cervical cancer cell line C-33A [14]. We examined whether peak 1 had this effect on U2OS cells (20 μg/mL) when compared to treatment with the chemotherapy drug doxorubicin. Cells treated with peak 1 displayed chromatin condensation and nuclear shrinkage, as observed by DAPI staining, similar to that seen after treatment with doxorubicin (Figure 6A). To confirm that the condensed chromatin and nuclear shrinking was due to apoptosis, we monitored the cleavage of PARP (115 kDa) and the appearance of the 85 kDa fragment (Figure 6B). A 85 kDa band was observed when U2OS cells were treated for 24 h with peak 1. Similarly, PARP cleavage was also observed after treatment of the cells with doxorubicin. These data indicate that peak 1 may be responsible for the apoptotic activity of X. aethiopica extract. The molecular mechanisms involved in this X. aethiopica-induced cell cycle arrest and apoptosis will require further investigation.

To determine unambiguously the chemical structure of the active compound corresponding to peak 1 of HPLC profile, we first analyzed our mass spectrometry data and found a molecular weight of 316, possibly indicating a C_20_H_28_O_3 _diterpene. We elucidated the structure by using standard and multi-dimensional NMR spectroscopy. Finally, we compared our ^1^H and ^13 ^C NMR data with those of the known compound ent-15-oxokaur-16-en-19-oic acid (EOKA) (Figure 2B) and proved to be identical to the literature data [17-19].

Taken together, our data demonstrate that EOKA is the main cytotoxic compound in extract of X. aethiopica.

## Competing interests

The authors declare that they have no competing interests.

## Authors' contributions

AC, AV and IT carried out the pharmacology and cell biology experiments. MD and SC performed the purification and the chemical characterization. VP, AP and TA inspirated and supervised the work of AC. AV and BD directed the work and wrote the manuscript. All authors read and approved the final manuscript.
